# Clinical Difficulties Related to Direct Composite Restorations: A Multinational Survey

**DOI:** 10.1016/j.identj.2024.06.012

**Published:** 2024-07-23

**Authors:** Anna Lehmann, Kacper Nijakowski, Jakub Jankowski, David Donnermeyer, João Carlos Ramos, Milan Drobac, João Filipe Brochado Martins, Ömer Hatipoğlu, Bakhyt Omarova, Muhammad Qasim Javed, Hamad Mohammad Alharkan, Olga Bekjanova, Sylvia Wyzga, Moataz-Bellah Ahmed Mohamed Alkhawas, Rutendo Kudenga, Anna Surdacka

**Affiliations:** a Department of Conservative Dentistry and Endodontics, Poznan University of Medical Sciences, Poznan, Poland; b Department of Periodontology and Operative Dentistry, University Münster, Münster, Germany; c Center for Innovation and Research in Oral Sciences (CIROS) and Institute of Operative Dentistry, Faculty of Medicine, University of Coimbra, Coimbra, Portugal; d Department of Dental Medicine, Faculty of Medicine, University of Novi Sad, Novi Sad, Serbia; e Departments of Endodontology Academic Centre for Dentistry Amsterdam, University of Amsterdam and Vrije Universiteit Amsterdam, Amsterdam, The Netherlands; f Department of Restorative Dentistry, Niğde Ömer Halisdemir University, Niğde, Turkiye; g Department of Therapeutic Dentistry, Kazakh National Medical University by S.D. Asfendiyarov, Almaty, Kazakhstan; h Department of Conservative Dental Sciences, College of Dentistry, Qassim University, Buraydah, Qassim, Saudi Arabia; i Department of Faculty Therapeutic Dentistry, Tashkent State Dental Institute, Tashkent, Uzbekistan; j Endodontic Department, Al-Azhar University, Cairo, Egypt; k Department of Odontology, University of Pretoria, Riviera, Pretoria, Republic of South Africa

**Keywords:** Composite resin, Permanent dental filling, Dental restoration failure, Clinical protocol, Questionnaire

## Abstract

**Aims:**

Composite materials are widely used in dentistry for direct tooth restorations. However, they are highly sensitive to the working technique employed during the restorative procedure. Even minor procedural errors can have a significant impact on the quality including the longevity of the restoration. Hence the aim of this study was to determine the material preferences and analyse the clinical problems associated with direct composite restorations in a cohort of dentists.

**Methods:**

A 20-item online questionnaire was created in English and administered 1830 general dentists and specialists in 13 countries. The first section of the questionnaire included four questions to elicit demographic data, and the second section comprised 16 questions focused on material preferences for conservative restorations, durability of composite restorations, and the most challenging stages the dentists faced during the composite restorative procedures.

**Results:**

Respondents decided most often to use composite materials for the tooth restorations (OR 997.4, 95% CI 233.8-4254.8, *P* value <.001). Most respondents indicated that the durability of composite restorations was approximately 7 to 10 years (41.5%). Among the factors affecting durability, maintenance of a dry cavity was the most often reported reason (47.1%) and the foremost challenge faced by dentists (61.0%) during the composite restorative procedures.

**Conclusions:**

Our study confirmed that resin-based composites are the most popular material for direct restoration in many countries. Although working with this material is difficult and involves multiple steps, maintaining a dry cavity during bonding, and material application may affect the therapeutic success and durability of these restorations. Clinicians need to be attentive to this issue and be prepared to adapt their decision-making and consider opting for alternative restorative materials, if appropriate.

## Introduction

### Significance of composite restorations

Resin-based composites (RBCs) have become the predominant choice for dental restorations, and are the most commonly used materials. Their popularity has increased owing to their functionality, aesthetics, and relatively low costs compared to indirect materials.^1^, ^2^, ^3^ Nowadays, dentists value the aesthetic properties and repairability of these materials but are also aware of polymerisation shrinkage and experience discolouration.^4^

However, in the field of restorative dentistry, numerous materials are used for tooth restoration that also serve as alternatives to composite materials such as compomers, glass ionomer cement (GIC), and resin-modified GIC (RMGIC).^5^, ^6^, ^7^

### Alternative materials and factors influencing material choice

GIC is a popular material in restorative dentistry because of its physical and chemical bonding to the tooth structure, especially dentin compared to RBCs, acceptable aesthetics, biocompatibility, sustained fluoride release, inhibition of bacterial acid activity, and ease of clinical application.^8^^,^^9^ However, traditional GICs present challenges such as dehydration, initial moisture sensitivity, extended setting time, acidification of the oral environment, and rough surface, potentially compromising the mechanical aspects of restoration.^10^^,^^11^

Compomers have been successfully used as direct restorative resins for various applications. This material, which is characterised as a polyacid-modified resin composite, combines the features of composites and GIC. Although they possess the handling properties of traditional RBCs and the fluoride-releasing characteristics of GICs, they encounter challenges such as brittleness, low durability, extended curing time, and water sensitivity, which limit their broader applications.^1^^,^^6^^,^^12^

Another type of combination featuring the characteristics of glass ionomers with composite resins, besides compomers, is RMGIC. While RMGICs have a monomer and undergo partial addition polymerisation, enhancing the acid-base process with controllable light activation, their physical properties resemble conventional GICs, albeit with slightly reduced biocompatibility.^13^^,^^14^ Moreover, similar to RBCs, they exhibit toxicity associated with the release of methacrylates.^14^^,^^15^

There are numerous possibilities, but the choice of material depends on various factors, including the dentists’ preferences and clinical needs.^16^^,^^17^ Nevertheless, composite materials are popular because of their multifunctionality.^2^^,^^3^^,^^18^

### Challenges in the composite application process

The composite application procedure comprises several stages, and the correct execution of these steps is crucial for the longevity of the restoration and clinical success. These procedure stages include initial cavity preparation, subsequent tooth tissue etching, and application of a compatible bonding system, followed by polymerisation, occlusal adjustment, and polishing of the previously placed RBC restoration.^19^, ^20^, ^21^, ^22^

During the polymerisation of composite materials (or other polymerising materials such as RMGIC), the material undergoes shrinkage (polymerisation shrinkage), which generates stress within the cavity.^1^^,^^4^^,^^23^ The magnitude of this stress, among other factors, depends on the configuration of the cavity, which is expressed by the configuration factor (c-factor), defined as the ratio of bonded surfaces to free surfaces. The stress within the cavity resulting from polymerisation shrinkage can lead to enamel deformation and fractures, damage to the adhesive bond between the tooth and the composite, and the formation of a gap, which may result in microleakage, postoperative sensitivity, and recurrent caries.^24^^,^^25^

Many adhesive systems are available on the market, categorised by generation, number of steps required for application, and etching method. Currently, eighth-generation single-step self-adhesive systems are in use; however, previous generations of adhesive systems are still being utilised. Depending on the generation and manufacturer, these systems differ in properties and bonding strength to dental tissues.^26^, ^27^, ^28^

Composite restorations are particularly preferred in the posterior segments because of the good mechanical strength of the composite material, and the anterior segment because of their aesthetics. However, to achieve the aforementioned characteristics, the precise execution of each of the steps listed above is required, which can be challenging. When placing a composite material, absolute dryness of the operative field is necessary, which is not always achievable in cases of deep subgingival cavities (with bleeding) or when restorations are placed in children (owing to the small operative field/oral cavity and difficulty in maintaining stillness). Additionally, adjusting the restoration to the occlusion can be more challenging, especially with extensive fillings on the occlusal surface, as even a slight excess of the composite material can cause discomfort to the patient. This issue is less pronounced with GIC restorations because of the lower hardness and greater wearability of the material, allowing such restorations to adapt to the occlusion in a relatively short time in areas that may have escaped the dentist's attention during the occlusal adjustment stage.^4^^,^^29^

Moreover, the polishing sequence is crucial, because a more intricate polishing procedure ensures the smoothness of the composite, enabling its proper functioning in the oral environment. Additionally, it contributes to the extended longevity and aesthetics of composites.^30^^,^^31^ This aesthetics is particularly significant for anterior tooth restoration; however, obtaining this requires adherence to the aforementioned direct restoration procedure, which is technique-sensitive and demands attention to various factors encountered during the process, particularly in posterior restorations.^22^^,^^30^^,^^32^ These factors may include maintaining dry conditions within the cavity,^1^^,^^8^^,^^22^ modelling tooth anatomy (owing to the frequent complexity and intricacy of grooves and fissures on the surfaces of posterior teeth), or even the subsequent correct restoration adjustment to occlusal conditions.^17^ Improper adaptation and finishing (polishing) of restorations may seriously affect patient well-being and restoration longevity.^17^^,^^21^

The complexity of the process and the need to consider many details during the placement of composite restorations have led to errors in the procedure. These shortcomings may lead to complications such as recurrent caries and irritating effects of the dentin-pulp complex or discolouration, significantly reducing restoration longevity and necessitating replacement.^32^, ^33^, ^34^, ^35^ Therefore, the application of composite restorations poses practical challenges for the operators. Each component in this process may influence the subsequent longevity of the restoration, thereby requiring high operator precision.

### Objectives

Our study offers insights into dentists’ preferences regarding the materials used for direct restorations as well as the difficulties that dentists consider their most challenging encounter during the application of direct composite restorations. Additionally, we focused on factors that influence the longevity of direct composite restorations based on the opinions of dentists.

## Material and methods

Polish investigators (A. L. and K. N.) designed the questionnaire for dental practitioners. This multinational study was conducted between May and November 2023. Researchers from 23 countries across all continents were invited to participate via email. However, only 13 researchers from 13 countries (Germany, Poland, Portugal, Serbia, the Netherlands, Turkey, Kazakhstan, Pakistan, Saudi Arabia, Uzbekistan, Egypt, the Republic of South Africa, and Canada) across four continents (Europe, Asia, Africa, and North America) responded and agreed to participate. Each collaborating researcher took responsibility for securing ethical approval in their country and, if necessary, ensuring strict adherence to country-specific ethical standards throughout the study (see: Conflict of interest – Ethics approval).

The final online questionnaire was created in English in May 2023. This form was also available in the native languages of Serbia and Turkiye. A 20-item questionnaire was administered to general dentists and specialists, particularly to those in conservative dentistry and endodontics. The unique survey URL link was disseminated across various social media channels and platforms, including dental associations specific to each country. Each researcher was sent at least two reminders to increase the response rate.

The questionnaire comprised two sections. The first section included four questions related to demographic characteristics (country, gender, work experience, and specialisation). The second section comprised 16 questions focused on material preferences for conservative restorations, estimated period, and the main factors for the durability of composite restorations, as well as the most problematic stages during the composite restoration procedure. Before the survey was disseminated, it was validated using a test-retest method. A cohort of 20 participants responded to these questions twice with a 2-week interval between responses. Intrarater agreement was evaluated using kappa statistics. The total kappa score assessed was 0.85. The questionnaire is attached as a Supplemental Material.

Statistical analyses were performed using the MedCalc Statistical Software version 22.014 (MedCalc Software Ltd.) and Statistica Software, version 13.3 (StatSoft). The results are presented as percentages of the respondents’ answers or odds ratios calculated separately for each country. Qualitative variables were compared using Pearson's chi-square test. Pooled odds ratios are reported in forest plots. Owing to the high values of *I*^2^, random effects were selected. The significance level was set at *α* = 0.05. For proper analyses, the five-level questions were binary categorised (‘never’ or ‘rare’ as ‘no’, ‘usually’, ‘often’ or ‘always’ as ‘yes’). Radar (spider) plots were visualised using Excel from Microsoft 365 (Microsoft Corporation).

## Results

A total of 1830 dentists from 13 countries participated in the survey. Most respondents were from Kazakhstan (*n* = 203) and Poland (*n* = 200), while the fewest were from the Netherlands (*n* = 56). There was a slight female predominance (52.8%). Nearly 1/3 of the respondents had more than 15 years of professional experience. Less than half of the respondents were nonspecialised dental practitioners, while more than 30% specialised in conservative dentistry. Table 1 provides detailed demographic data.

**Table 1:** Detailed demographic data about respondents (*n* = 1830).

	*n*	%
Country		
Germany	138	7.5
Poland	200	10.9
Portugal	118	6.4
Serbia	154	8.4
the Netherlands	56	3.1
Turkiye	173	9.5
Kazakhstan	203	11.1
Pakistan	153	8.4
Saudi Arabia	138	7.5
Uzbekistan	191	10.4
Canada	100	5.5
Egypt	100	5.5
Republic of South Africa	106	5.8
Gender		
Female	967	52.8
Male	863	47.2
Work experience		
<6 y	667	36.5
6-15 y	567	31.0
16-25 y	306	16.7
>25 y	290	15.8
Specialisation		
No specialisation	823	45.0
Conservative dentistry/endodontics	601	32.8
Periodontology/oral surgery/maxillofacial surgery	136	7.4
Paediatric dentistry	112	6.1
Prosthodontics/orthodontics	133	7.3
Radiology or other	25	1.4

**Fig. 1:**
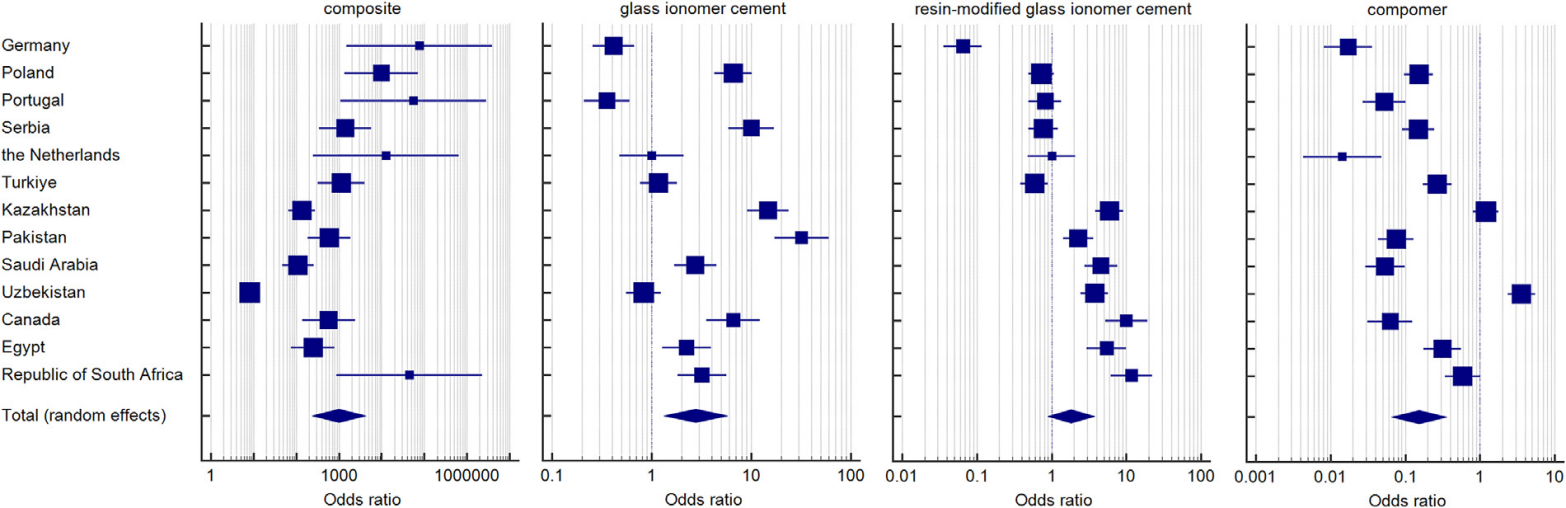
Forest plots presenting the pooled odds ratios for preferences about choice of dental materials for direct restorations: (A) composite, (B) glass ionomer cement, (C) resin-modified glass ionomer cement, (D) compomer.

Respondents decided most often to use composite materials for the tooth restorations (OR 997.4, 95% CI 233.8-4254.8, *P* value <.001) – Figure 1A. Among the surveyed countries, European countries such as Germany (OR 76,729, 95% CI 1512-3894,611), Portugal (OR 56,169, 95% CI 1105-2854,427), the Netherlands (OR 12,769, 95% CI 249-654,798) and Poland (OR 9801, 95% CI 1367-70,271), and the Republic of South Africa (OR 45,369, 95% CI 892-2307,731), most frequently chose composite restorations. Composites were relatively seldom chosen in Uzbekistan (OR 7.952, 95% CI 5.039-12.550). The second most widely used material was GIC (OR 2.766, 95% CI 1.323-5.786, *P* value .007), especially the most commonly used by Pakistanis (OR 31.947, 95% CI 17.066-59.806), Kazakhs (OR 14.694, 95% CI 9.090-23.754), Serbs (OR 9.999, 95% CI 5.928-16.866) and Poles (OR 6.612, 95% CI 4.273-10.231) – Figure 1B. However, it was rare among the Germans (OR 0.413, 95% CI 0.255-0.670) and Portuguese (OR 0.354, 95% CI 0.209-0.599). Similarly, RMGIC was chosen more often in non-European countries (Figure 1C), especially RSA (OR 11.674, 95% CI 6.135-22.212), Canada (OR 10.028, 95% CI 5.240-19.190) and Kazakhstan (OR 5.957, 95% CI 3.881-9.143). It was least popular in Germany (OR 0.065, 95% CI 0.036-0.117). By far, the least preferred material for restorations was compomer (OR 0.153, 95% CI 0.065-0.357, *P* value <.001) (Figure 1D). Indeed, this material was significantly more frequently selected in Uzbekistan (OR 3.587, 95% CI 2.353-5.469). The detailed results for each country are presented in Table 2.

**Table 2:** The pooled odds ratios describing preferences about choice of dental materials for direct restorations.

Country	Composite			Glass ionomer cement			Resin-modified glass ionomer cement			Compomer		
	OR	95% CI	Weight	OR	95% CI	Weight	OR	95% CI	Weight	OR	95% CI	Weight
Germany	76,729	1511.663-3894,611.348	5.50	0.413	0.255-0.670	7.75	0.065	0.036-0.117	7.62	0.017	0.008-0.036	7.56
Poland	9801	1366.989-70,270.954	7.88	6.612	4.273-10.231	7.80	0.726	0.490-1.075	7.84	0.151	0.098-0.234	7.86
Portugal	56,169	1105.285-2854,426.825	5.50	0.354	0.209-0.599	7.70	0.816	0.489-1.360	7.72	0.053	0.027-0.101	7.66
Serbia	1406.25	345.294-5727.123	8.49	9.999	5.928-16.866	7.71	0.771	0.493-1.206	7.79	0.150	0.091-0.247	7.81
the Netherlands	12,769	249.004-654,798.324	5.49	1.000	0.477-2.098	7.42	1.000	0.477-2.098	7.40	0.014	0.004-0.048	6.92
Turkiye	1128.96	320.899-3971.807	8.63	1.176	0.771-1.793	7.81	0.586	0.383-0.896	7.81	0.268	0.172-0.418	7.85
Kazakhstan	136.598	66.356-281.193	9.02	14.694	9.090-23.754	7.75	5.957	3.881-9.143	7.81	1.194	0.809-1.763	7.89
Pakistan	600.25	189.214-1904.197	8.71	31.947	17.066-59.806	7.58	2.275	1.439-3.595	7.78	0.076	0.044-0.130	7.77
Saudi Arabia	110.25	47.720-254.717	8.95	2.735	1.681-4.451	7.75	4.564	2.751-7.573	7.72	0.054	0.030-0.099	7.71
Uzbekistan	7.952	5.039-12.550	9.14	0.828	0.554-1.237	7.83	3.758	2.461-5.738	7.81	3.587	2.353-5.469	7.87
Canada	576	139.994-2369.930	8.48	6.612	3.566-12.259	7.59	10.028	5.240-19.190	7.54	0.063	0.031-0.125	7.62
Egypt	245.444	76.394-788.580	8.70	2.250	1.278-3.962	7.66	5.444	2.973-9.969	7.60	0.316	0.178-0.564	7.74
Republic of South Africa	45,369	891.935-2307,731.198	5.50	3.202	1.827-5.614	7.66	11.674	6.135-22.212	7.55	0.588	0.341-1.012	7.77
Total (random effects): OR and *P* value	997.391	233.802-4254.840	<0.001	2.766	1.323-5.786	0.007	1.823	0.887-3.747	0.102	0.153	0.065-0.357	<0.001

**Fig. 2:**
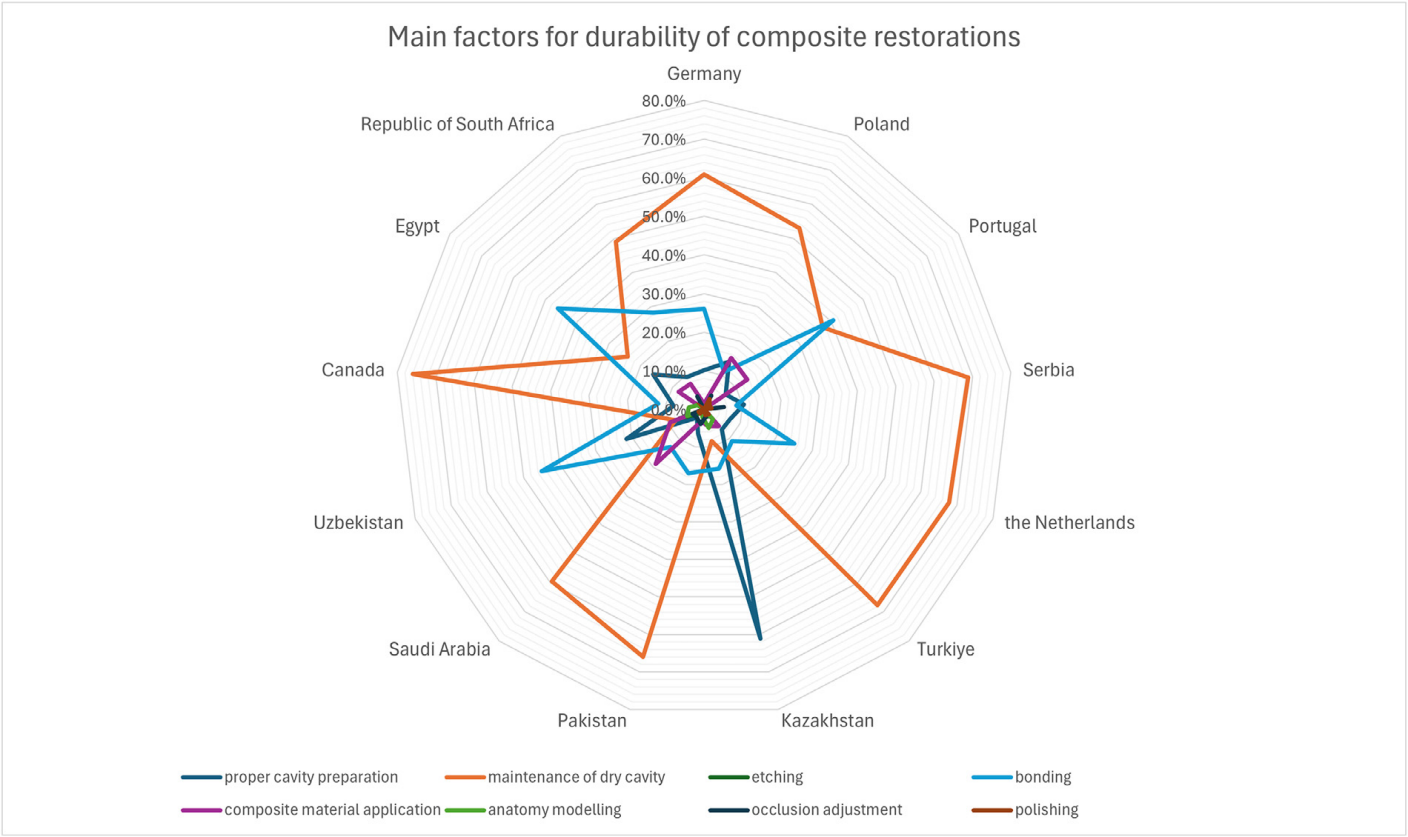
Main factors for durability of direct composite restorations depending on country (*P* value <.001 for Pearson's chi-square test).

Among the factors affecting the durability of composite restorations, maintenance of the dry cavity was most often reported (47.1%). The next key step was bonding (22.0%), indicated most often by the Uzbeks, Egyptians, and Portuguese, and proper cavity preparation (16.1%), which was the most common response among the Kazakhs (Figure 2). The most frequently reported factors differed significantly between countries (*P* value <.001, Pearson's chi-square test).

Regarding the estimated durability of the composite restorations (Table 3), most respondents indicated 7 to 10 years (41.5%), especially from the Netherlands (57.1%) and Poland (55.0%). The next most common period was 3 to 6 years (32.3%), followed by Serbs (52.6%), Pakistan (47.1%), and Kazakhs (45.8%). The shortest durability (<3 years) was most often indicated by Uzbeks (14.1%) and Pakistanis (12.4%), and the longest durability (>15 years) by Canadians (12.0%), Turks (11.0%), and Dutch (10.7%). Similarly, significant differences in answer distribution were observed when considering work experience (Table 4). Regardless of work experience, the most common responses were 7 to 10 years and 3 to 6 years. In the group of dentists with the longest experience, an interval above 15 years of restoration durability was indicated much more often compared to the rest of the respondents. In addition, the shortest durability (up to 3 years) was indicated by dentists with the shortest experience.

**Table 3:** Estimated longevity of direct composite restorations depending on country (*P* value <.001 for Pearson's chi-square test).

Country	All	<3 y		3-6 y		7-10 y		11-15 y		>15 y	
Germany	138	0	0.0%	28	20.3%	60	43.5%	36	26.1%	14	10.1%
Poland	200	4	2.0%	58	29.0%	110	55.0%	24	12.0%	4	2.0%
Portugal	118	2	1.7%	26	22.0%	60	50.8%	18	15.3%	12	10.2%
Serbia	154	6	3.9%	81	52.6%	48	31.2%	13	8.4%	6	3.9%
the Netherlands	56	2	3.6%	6	10.7%	32	57.1%	10	17.9%	6	10.7%
Turkiye	173	9	5.2%	56	32.4%	64	37.0%	25	14.5%	19	11.0%
Kazakhstan	203	10	4.9%	93	45.8%	73	36.0%	19	9.4%	8	3.9%
Pakistan	153	19	12.4%	72	47.1%	47	30.7%	7	4.6%	8	5.2%
Saudi Arabia	138	6	4.3%	30	21.7%	66	47.8%	24	17.4%	12	8.7%
Uzbekistan	191	27	14.1%	56	29.3%	64	33.5%	25	13.1%	19	9.9%
Canada	100	0	0.0%	20	20.0%	44	44.0%	24	24.0%	12	12.0%
Egypt	100	4	4.0%	38	38.0%	48	48.0%	4	4.0%	6	6.0%
Republic of South Africa	106	12	11.3%	28	26.4%	44	41.5%	20	18.9%	2	1.9%
Total	1830	101	5.5%	592	32.3%	760	41.5%	249	13.6%	128	7.0%

**Table 4:** Estimated longevity of direct composite restorations depending on work experience (*P* value <.001 for Pearson's chi-square test).

Work experience	All	<3 y		3-6 y		7-10 y		11-15 y		>15 y	
<6 y	667	56	8.4%	243	36.4%	279	41.8%	62	9.3%	27	4.1%
6-15 y	567	23	4.0%	185	32.6%	230	40.6%	86	15.2%	43	7.6%
16-25 y	306	9	2.9%	75	24.5%	147	48.0%	51	16.7%	24	7.9%
>25 y	290	13	4.5%	89	30.7%	104	35.9%	50	17.2%	34	11.7%
Total	1830	101	5.5%	592	32.3%	760	41.5%	249	13.6%	128	7.0%

**Fig. 3:**
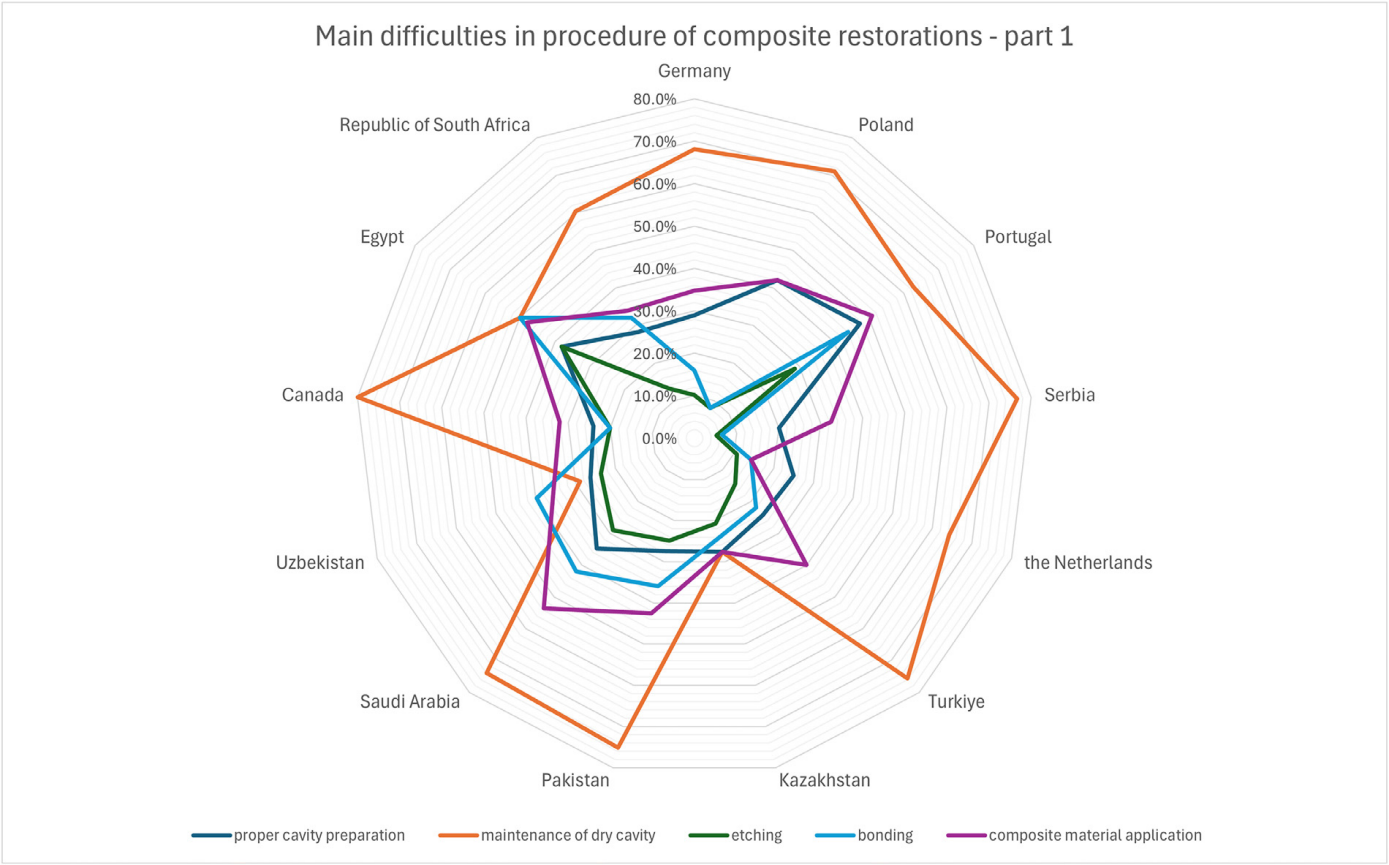
Main difficulties in procedure of direct composite restorations depending on country – part 1 (*P* value <.001 for Pearson's chi-square test).

Based on Figure 3, maintenance of the dry cavity was considered the most problematic initial stage of restoration preparation in all countries (61.0%), except Uzbekistan, where bonding and composite material application were the most common problems (39.8% and 35.6%, respectively). In Egypt, maintaining a dry cavity was as problematic as bonding (each 50.0%), and in Kazakhstan, proper cavity and composite material preparation were problematic (27.6%). In general, the least problematic step was etching (18.6%).

**Fig. 4:**
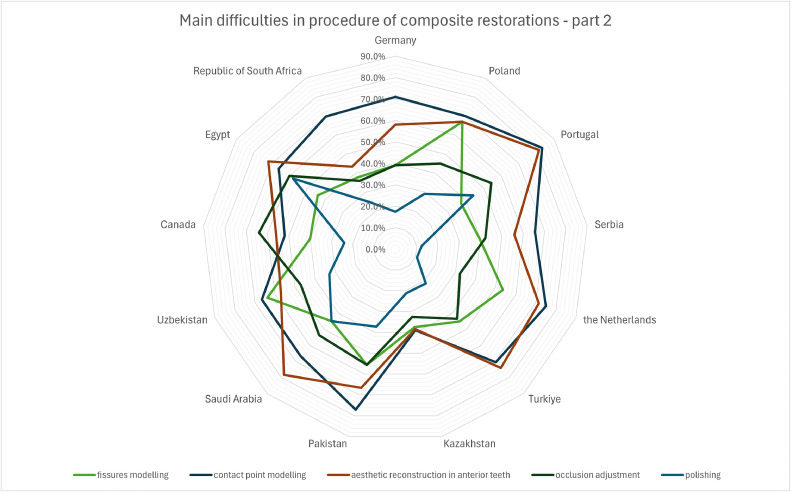
Main difficulties in procedure of direct composite restorations depending on country – part 2 (*P* value <.001 for Pearson's chi-square test).

Among the further steps of composite restorations (Figure 4), contact point modelling and aesthetic reconstruction of the anterior teeth were most commonly reported as problematic in most countries (66.1% and 62.0%, respectively). However, Canadians reported the biggest problem with occlusion adjustment (64.0%). The least problematic aspect seemed to be the final polishing of the restoration (28.9%), which was only sometimes ahead of fissure modelling, as in Egypt and Portugal. For all the described difficulties, the differences in responses from different countries were significant (*P* value <.001, Pearson's chi-square test).

## Discussion

A questionnaire study is the best way to examine the choices dentists make in their offices and the difficulties they face in their daily work. Our study is innovative because most previous surveys on RBCs focused on the durability, repair, or replacement of restorations.^36^, ^37^, ^38^ Our respondents strongly indicated that they preferred composite materials for direct reconstruction, similar to the studies by Kopperud et al.^39^ and the newest one by Rafique et al.^40^

This result corresponded to the global trend observed over several decades.^41^^,^^42^ This is because the field of dentistry has shifted away from amalgam restorations. Many developed countries have banned or are in the process of restricting its use. Recent studies have shown that the possibility of repairing composite restorations is crucial for dentists. It helps to avoid enlarging the cavity during replacement.^43^ However, it is important to note that the choice of material for reconstruction depends significantly on the clinical situation, and the survey was independent of this.

The second most commonly chosen materials were GICs. This choice seems excellent, considering that this material can be a panacea for ubiquitous recurrent caries, in which RBCs have a large share.^44^^,^^45^ Functional properties such as biocompatibility, fluoride release, natural adhesion to dentin and enamel, and moisture tolerance during the procedure can easily compete with those of aesthetic composite materials. The preferred material was a combination of glass ionomers and composite resins – RMGIC. Research indicates that GICs have better properties than RMGICs. Our survey results showed that dentists know the characteristics and properties of materials.^46^ Our findings correlate with those of Rafique et al.^40^ in that dentists strongly prefer GICs to RMGICs.

The last indicated material, the compomer, seems to be a poorly known and widespread type of restoration among dentists. Similar results were obtained in a survey conducted by Drachev et al.^47^ The respondents indicated their preferences as follows: composite, GIC, RMGIC, a combination of GIC and composite (sandwich technique), and compomers.

Our respondents indicated that the durability of direct composite restorations ranged from 7 to 10 years. These findings are consistent with data obtained by other researchers, showing that the most frequently indicated durability ranges from 3 to 10 years.^48^^,^^49^ In our questionnaire, the question about durability did not include detailed information on the extent of the defect, which may be a limitation. However, the most recent studies by Bresser et al. and Josic et al.^49^^,^^50^ showed no difference between extensive composite restoration and indirect reconstruction, which may be surprising.

For many years, reports on the sensitivity of composites to insertion techniques or operator-related factors have become increasingly common in the literature.^29^^,^^51^ In our survey, dentists most often related the durability of the restoration to maintaining dryness during the material application procedures. Similar results have been reported by Kopperud et al.^39^ Remnants of blood and saliva proteins can impair the adhesion between the adhesive and composite layers. In addition, rinsing with contaminants after adhesive application may disrupt the oxygen-inhibited and unpolymerised layers.^52^ Challenges related to maintaining dryness during cavity restoration require a rubber dam, which increases the time of visit and cost and cannot be used by everyone.^53^

Dentists need to choose materials that are more resistant to moisture during pregraduate education and later in postgraduate courses. For example, in restorations on proximal surfaces, the open sandwich technique allows the GIC layer to be exposed to the oral environment (as opposed to the closed sandwich technique, in which the composite material fully covers the GIC layer).^54^^,^^55^ Unfortunately, as already mentioned, economic factors often come into play in the office, and neither the doctor nor the patient is willing to make a second appointment for the same tooth. The pressure to complete the reconstruction in one visit becomes more important than the prognosis and the condition of the tooth and periodontium.

Bonding is another critical step in composite restorations. Research has shown that the bond is the weakest link in the composite, and there should be as little of it as possible; when used in excess, it creates a discoloured borderline.^56^^,^^57^ In studies by Brunton et al. and Kattan et al.,^38^^,^^58^ dentists most often indicated these noncarious marginal defects as an indication for repair or even replacement of the entire restoration.

In our study, contact point modelling and aesthetic reconstruction of anterior teeth were reported to be problematic in most countries (66.1% and 62.0%, respectively). Establishing proper contact with composites in Class II restorations is a major problem. Various restoration techniques and matrix systems have been introduced to overcome this problem.^59^^,^^60^ Parpaiola et al.^37^ indicated that as many as 50% of composite restorations had unsatisfactory restoration anatomy, corresponding to our survey results. This is further proof of the difficulty and technical demand of the RBC.

Similarly, postoperative sensitivity was significantly associated with the use of direct posterior composites. A 50%prevalence rate of postoperative sensitivity has been reported in the literature. Class II restorations in posterior teeth are mainly associated with the aforementioned problem.^37^^,^^59^^,^^61^

The last mentioned problem seems to be the final polishing of the restoration (28.9%), and it has been proven that this is a crucial step influencing therapeutic success in the case of composite material.^30^^,^^62^ Appropriate polishing can hide imperfections such as imperfect anatomy or colour restoration. However, this stage of reconstruction may be marginalised or even skipped.

### Limitations

Among the limitations of our study, we emphasised some potential biases. We did not indicate to respondents the clinical situation for choosing the restorative material. We also did not consider sociodemographic factors, including patient expectations or healthcare system requirements.

The original online questionnaire was available in English (except in two countries), which generally limited its accessibility to English speakers. Thus, the results may not fully reflect the dentists’ preferences and experiences in their respective countries. It may also be feared that dentists interested in conservative dentistry and younger practitioners who use social media will participate more frequently. Moreover, owing to the method of dissemination, the response rate to the survey could not be evaluated, weakening the study's validity. The number of dentists who differed between the participating countries may be a limitation. However, it should be noted that representatives of the medical profession do not seem willing to participate in the survey research, and the international cooperation network that we are creating is still being developed. We hope to expand the scope of this cooperation for further research.

## Conclusions

Dentists often choose composite materials; however, they experience numerous clinical difficulties while working with them. Therefore, they should be aware of these difficulties and manage them as effectively as possible. The reconstruction procedure using a composite material is a difficult multistage procedure. Maintaining a dry cavity is considered the most crucial stage of the composite restoration procedure. As dentists gain experience, they have noticed that creating durable composite restorations is the most challenging issue. However, this knowledge comes from the experience and observation of the effects of their work on patients.

## CRediT authorship contribution statement

**Anna Lehmann:** Conceptualization, Data curation, Formal analysis, Investigation, Methodology, Writing – original draft. **Kacper Nijakowski:** Conceptualization, Data curation, Formal analysis, Investigation, Methodology, Writing – original draft, Visualization, Writing – review & editing. **Jakub Jankowski:** Data curation, Methodology, Investigation, Writing – original draft. **David Donnermeyer:** Investigation. **João Carlos Ramos:** Investigation. **Milan Drobac:** Investigation. **João Filipe Brochado Martins:** Investigation. **Ömer Hatipoğlu:** Investigation. **Bakhyt Omarova:** Investigation. **Muhammad Qasim Javed:** Investigation. **Hamad Mohammad Alharkan:** Investigation. **Olga Bekjanova:** Investigation. **Sylvia Wyzga:** Investigation. **Moataz-Bellah Ahmed Mohamed Alkhawas:** Investigation. **Rutendo Kudenga:** Investigation. **Anna Surdacka:** Supervision, Writing – review & editing.

## Conflict of interest

The authors declare that they have no known competing financial interests or personal relationships that could have appeared to influence the work reported in this article.

## Appendix Supplementary materials

Image, application 1

## Footnotes

Supplementary material associated with this article can be found, in the online version, at doi:10.1016/j.identj.2024.06.012.
