# Supplementary material for: Clinical Difficulties Related to Direct Composite Restorations: A Multinational Survey
Source: Int Dent J. 2024 Jul 23;75(2):797–806. doi: 10.1016/j.identj.2024.06.012 (PMC11976477; doi:10.1016/j.identj.2024.06.012)
Supplement: Supplementary file 1 [file mmc1.docx]

Country:

1. How long have you been practicing dentistry?

Up to 5 years

6-15

16-25

Over 26 years

2. Specialization:

No specialization

Conservative dentistry/Endodontics

Pediatric dentistry

Prosthodontics/Orthodontics

Periodontology/Oral and maxillofacial surgery

Oral and maxillofacial radiology/Other

3. How often do you use the following materials for direct fillings?

Never 1 2 3 4 5 Always

Composite

Compomer

Resin-modified glass ionomer cement

Glass ionomer cement

4. In your opinion, what is average durability of direct composite filling?

Up to 3 years

3-6 years

7-10 years

11-15 years

More than 15 years

5. What stage of restorative procedure is, in your opinion, most crucial for the durability of direct composite filling?

a. proper cavity preparation

b. maintenance of dry cavity

c. etching

d. bonding

e. composite material application

f. anatomy modelling

g. occlusion adjustment

h. polishing

6. How problematic are the following stages of composite restoration in your practice?

No problem 1 2 3 4 5 Always problematic

a. proper cavity preparation

b. maintenance of dry cavity

c. etching

d. bonding

e. composite material application

f. fissures modelling

g. contact point modelling

h. aesthetic reconstruction in the anterior tooth

i. occlusion adjustment

j. polishing

Thank you for completing the survey
